# Supplementary material for: A chromosome-scale genome assembly and evaluation of mtDNA variation in the willow leaf beetle Chrysomela aeneicollis
Source: G3 (Bethesda). 2023 May 13;13(7):jkad106. doi: 10.1093/g3journal/jkad106 (PMC10320752; doi:10.1093/g3journal/jkad106)
Supplement: jkad106_Supplementary_Data [file jkad106_supplementary_data.zip › Supplemental_Figure_Legend_G3-2023-404236.docx]

**SUPPLEMENTAL FIGURE CAPTIONS.**

**Figure S1.** A) Karyoplot of 21 linkage groups and the proportion of masked repetitive sequence along the genome (blue, 50 kb windows) with gene density (high to low) shown as a white:green:black heatmap in 500 kb genomic intervals. B) Repetitive sequence distributions across the genome assembly plotted as the percentage of bases masked in 50 kb windows per repetitive sequence type. Regions found to be enriched for repeats (defined as at least two consecutive windows ≥ 2× the genome-wide average) highlighted with red boxes.

**Figure S2.** A) Region bracketing the cytochrome oxidase gene with tRNA molecules that differ between Big Pine Creek and Rock Creek. B) Predicted secondary structure of the tRNA^Leu^ alleles that differ between Big Pine Creek and Rock Creek. C) Predicted secondary structure of the tRNA^Lys^ alleles that differ between Big Pine Creek and Rock Creek.

**Figure S3.** A) Predicted secondary structure of the large ribosomal subunit gene for individuals possessing the northern mitochondrial genotype found in Rock Creek. B) Predicted secondary structure of the large ribosomal subunit gene for individuals possessing the mitochondrial genotype found in Big Pine Creek.
