# Supplementary figures and images for: A chromosome-scale genome assembly and evaluation of mtDNA variation in the willow leaf beetle Chrysomela aeneicollis
Source: G3 (Bethesda). 2023 May 13;13(7):jkad106. doi: 10.1093/g3journal/jkad106 (PMC10320752; doi:10.1093/g3journal/jkad106)

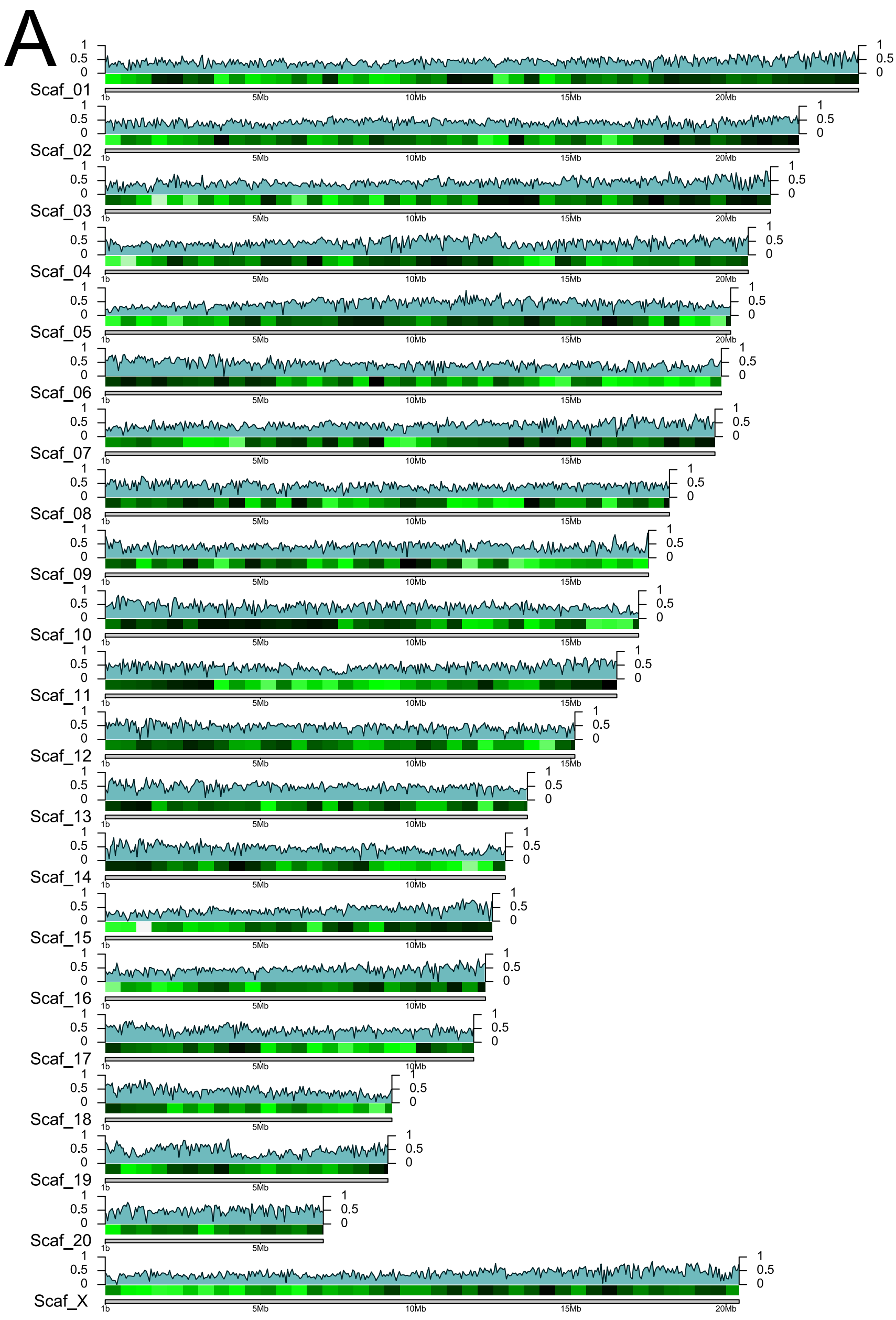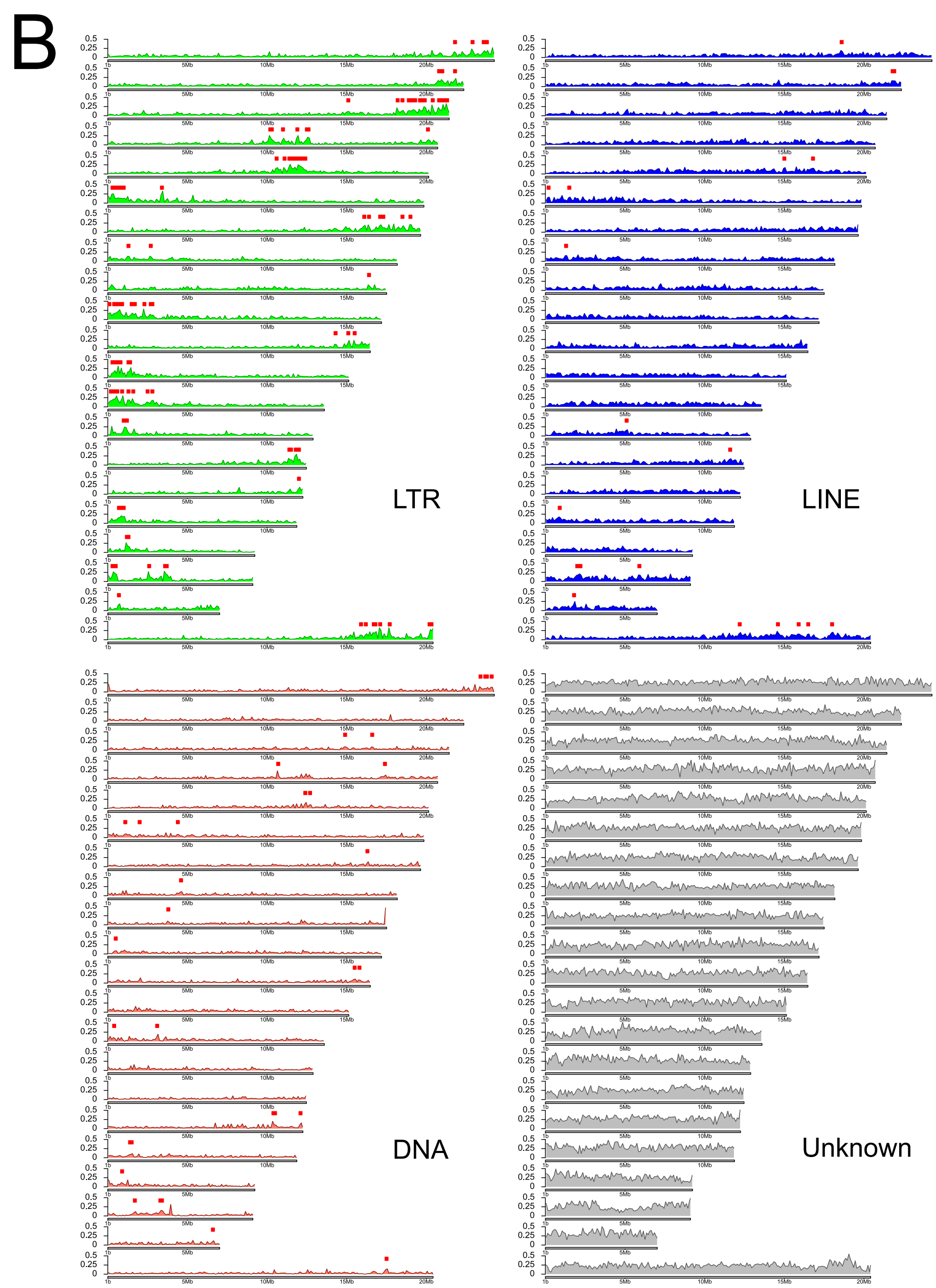

Supplement: jkad106_Supplementary_Data [file jkad106_supplementary_data.zip › Figure_S1_G3-2023-404236.pdf]

A.

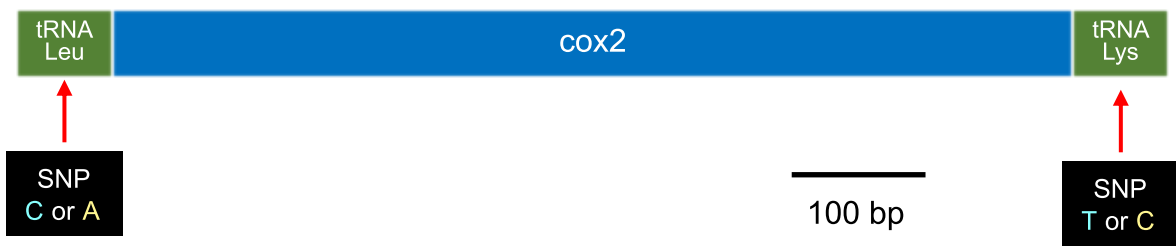

B.

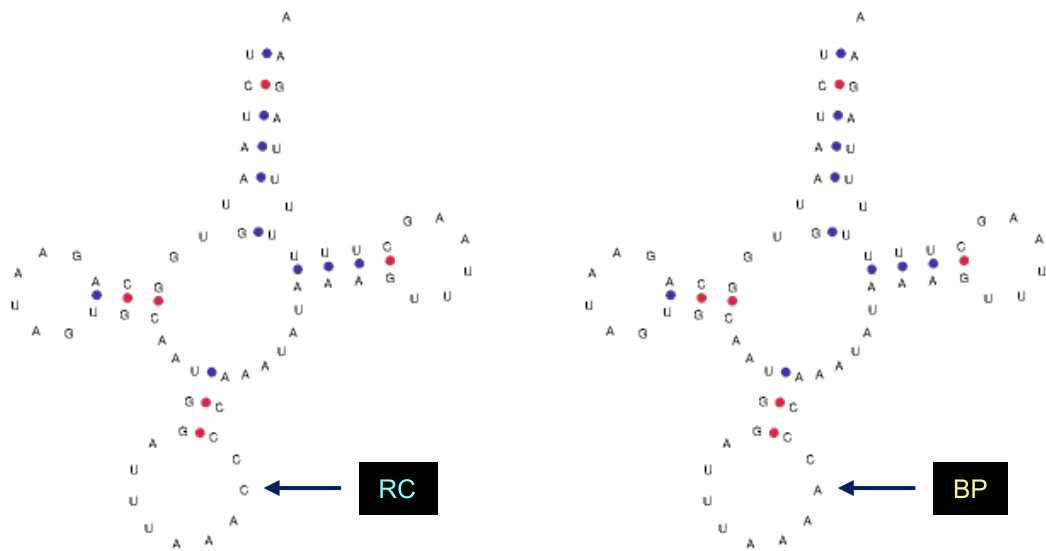

C.

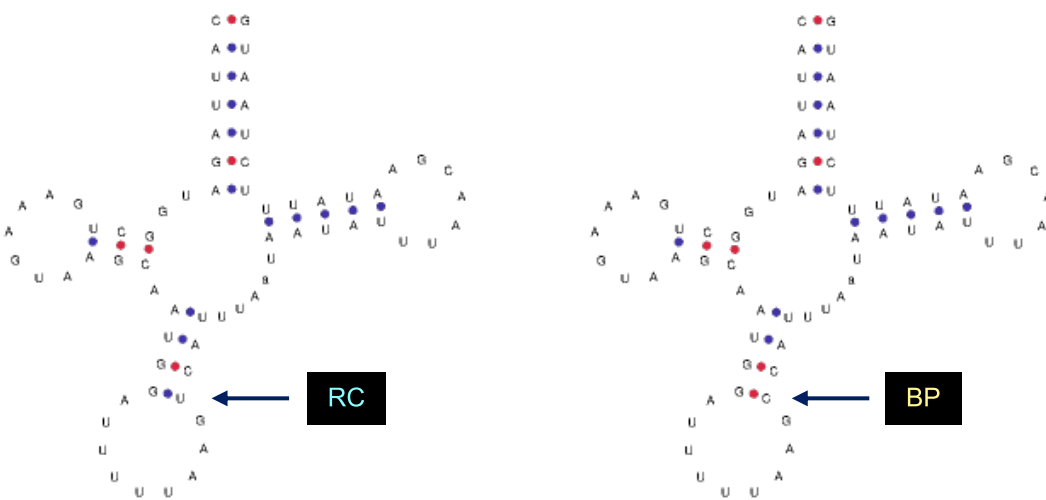

Supplement: jkad106_Supplementary_Data [file jkad106_supplementary_data.zip › Figure_S2_G3-2023-404236.pdf]

A. Rock Creek Free energy = -253.7 kcal/mol

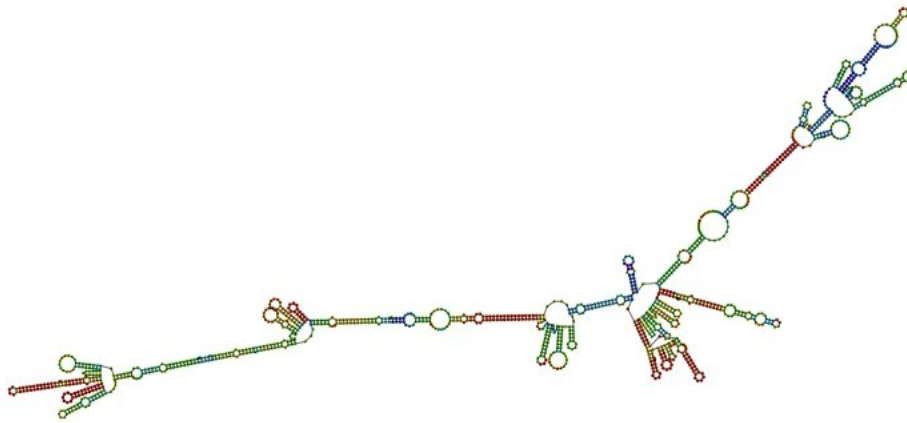

B. Big Pine Creek Free energy = -254.9 kcal/mol

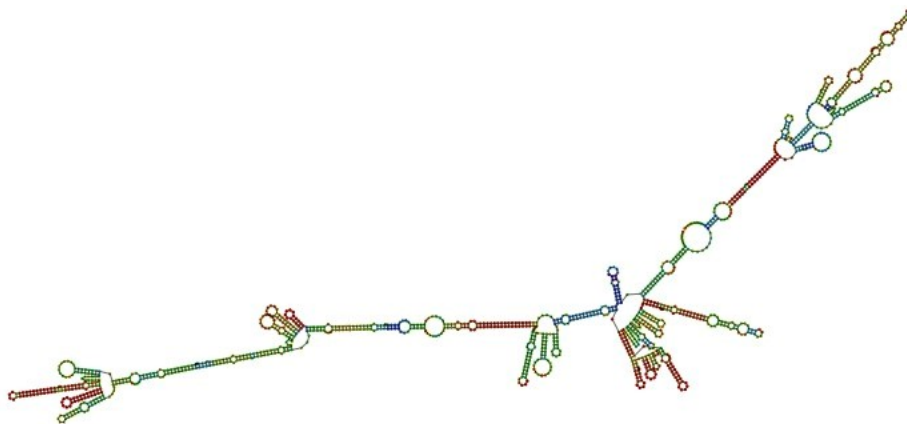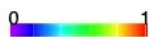

Supplement: jkad106_Supplementary_Data [file jkad106_supplementary_data.zip › Figure_S3_G3-2023-404236.pdf]
